# A Comparative Analysis of the Roles of von Willebrand Factor and ADAMTS13 in Hepatocellular Carcinoma: A Bioinformatics and Microarray-Based Study

**DOI:** 10.3390/cimb47040270

**Published:** 2025-04-10

**Authors:** Durmuş Ayan, Şerife Buket Bozkurt Polat, Ergül Bayram, Esma Özmen, Fatma Esin Aydın, Serpil Ersan

**Affiliations:** 1 Faculty of Medicine, Medical Biochemistry, Nigde Omer Halisdemir University, 51240 Nigde, Türkiye; buketbozkurt@yahoo.com (Ş.B.B.P.); ozmenesma07@gmail.com (E.Ö.); serpilersan@gmail.com (S.E.); 2 Medical Biochemistry Nigde, Nigde Omer Halisdemir University Research and Training Hospital, 51100 Nigde, Türkiye; eyaylagul@hotmail.com; 3 Faculty of Medicine, Medical Microbiology, Nigde Omer Halisdemir University, 51240 Nigde, Türkiye; esinaydin@ohu.edu.tr

**Keywords:** bioinformatics, hepatocellular cancer, VWF, ADAMTS13

## Abstract

Genetic and epigenetic alterations of various biomolecules at the molecular level can contribute to the pathogenesis of hepatocellular carcinoma (HCC) and negative impact prognosis. In this study, we aimed to investigate the effects of von Willebrand factor (VWF) and *ADAMTS13* on HCC prognosis, using bioinformatics tools. “These tools included GEPIA2, TIMER2, UALCAN database, KM-Plotter, TNM-plot, STRING, ENCORI, Human Protein Atlas, Targetscan 8.0, miRDB, Enrichr-KG, lncRNADisease and, GEO”. VWF expression levels were significantly upregulated in liver hepatocellular carcinoma (LIHC) tissues compared to healthy adjacent tissues. Conversely, ADAMTS13 expression levels were significantly downregulated in LIHC tissues compared with healthy adjacent tissues in GEPIA2 database. The upregulated expression of VWF was significantly associated with longer overall survival (OS). However, the downregulated expression of *ADAMTS13* was not significantly related to OS. The promoter regions of *VWF* and *ADAMTS13* were significantly hypomethylated. While a significant negative correlation was observed between VWF with CD4 + T cells, there was a positive correlation between VWF with CD8+ T cells. *ADAMTS13* expression positively correlated with CD4+ T cells. Additionally, a positive correlation was observed between *ADAMTS13* expression and long non-coding RNAs (lncRNAs) (H19, HOTAIR, MALAT1, and UCA1). Conversely, a negative correlation was observed between *VWF* expression and lncRNAs (H19, HOTAIR, MALAT1, and UCA1). Although these results are promising, they highlight the complexity of the interplay between *VWF* and *ADAMTS13* in HCC progression. According to microarray data, while VWF expression levels were significantly downregulated, ADAMTS13 expression levels were significantly upregulated in HCC compared with the control in the GEO database. Further studies are needed to elucidate the mechanisms underlying these markers.

## 1. Introduction

Hepatocellular carcinoma (HCC) is the most common form of liver cancer and it is associated with high mortality rates [1]. HCC is the third leading cause of cancer-related mortality globally, with a 5-year survival rate of approximately 18% [2].The mechanisms underlying HCC development are still not fully understood [3].

Rapid advancements in epigenetics have introduced a new perspective for uncovering the mechanisms underlying hepatocarcinogenesis, including DNA methylation, histone modifications, and chromatin remodeling [4,5]. These epigenetic alterations, which are linked to HCC progression and metastasis, are promising targets for biomarker development because of their reversible nature [6]. Understanding these mechanisms is essential to improve survival rates and predict treatment outcomes [6,7].

Recent studies have suggested a potential link between von Willebrand factor (*VWF)* and A disintegrin and metalloproteinase with a thrombospondin type 1 motif, member 13 (ADAMTS13) in the development and progression of HCC [8,9,10]. VWF is a glycoprotein that plays a crucial role in hemostasis, whereas ADAMTS13 is a metalloproteinase involved in the regulation of extracellular matrix components. These two proteins have been implicated in various cellular processes including angiogenesis, cell migration, and tumor growth [11,12]. Angiogenesis plays a crucial role in the progression of HCC. An imbalance in the VWF antigen (VWF:Ag)/ADAMTS13 activity (ADAMTS13:AC) ratio was reported to be associated with angiogenesis and hypercoagulability as well as prognosis in patients with various types of cancer, especially those receiving chemotherapy [8,13].

Understanding the relationship between *VWF* and *ADAMTS13* in HCC could provide valuable insights into the mechanisms underlying tumor development and progression [9]. Several findings suggest that the expression levels of both *VWF* and *ADAMTS13* are altered in HCC [14,15]. For example, high levels of *VWF* have been detected in HCC tissues compared to non-cancerous liver tissues, while elevated levels of ADAMTS13 may be observed following hepatic arterial infusion chemotherapy (HAIC) [8]. Factors such as impaired liver function, chronic liver inflammation, fibrosis, cirrhosis, increased systemic inflammation, tumor-associated cytokines (e.g., IL-6, TNF-α), tumor hypoxia, and elevated VWF levels in HCC may also contribute to a relative deficiency of ADAMTS13. This deficiency can lead to the development of a prothrombotic and immunosuppressive tumor microenvironment [16].

Long non-coding RNAs (lncRNAs) and microRNAs (miRNAs) are critical regulators of gene expression and play pivotal roles in the pathogenesis of HCC [17]. lncRNAs, typically longer than 200 nucleotides, function through diverse mechanisms such as acting as molecular scaffolds, sponges, or guides for chromatin remodeling complexes [17]. Their dysregulation in HCC has been associated with key processes such as tumor growth, metastasis, angiogenesis, and immune evasion [17]. miRNAs are small non-coding RNAs that regulate gene expression by binding to the target mRNAs, undergoing a multistep biogenesis processes involving transcription, processing, nuclear export, and incorporation into the RNA-induced silencing complex (RISC) for mRNA degradation or translational repression. miRNAs play a crucial role in HCC by regulating gene expressions involved in tumor initiation, progression, metastasis, and drug resistance. Dysregulated miRNAs can function as oncogenes (e.g., miR-21, miR-221) or tumor suppressors (e.g., miR-122, miR-199a) in HCC by modulating key signaling pathways such as Wnt/β-catenin, PI3K/AKT, and TGF-β. Moreover, extracellular vesicle-associated miRNAs serve as potential noninvasive biomarkers for HCC diagnosis, prognosis, and therapy response prediction [18,19]. ADAMTS13 has been recognized as a tumor-suppressive circular RNA that functions as a sponge for miR-484, though its specific target gene remains unidentified [20]. Among the mRNAs, *ADAMTS13*, which was upregulated, showed a correlation with the majority of lncRNAs [21]. The KCNQ1OT1-hsa-miR-24-3p-VWF competitive endogenous RNA (ceRNA) network was identified as a significant factor in the development of acute traumatic coagulopathy [22]. Nevertheless, the roles of lncRNA and miRNAs in gene regulation remain enigmatic.

Considering the above information, in this study, the possible imbalance between *VWF* and *ADAMTS13* in HCC was investigated using bioinformatics databases and microarray data, and the potential of the spectacular imbalance between *VWF* and *ADAMTS13* in HCC as a therapeutic agent was evaluated.

## 2. Materials and Methods

**Figure 1:**
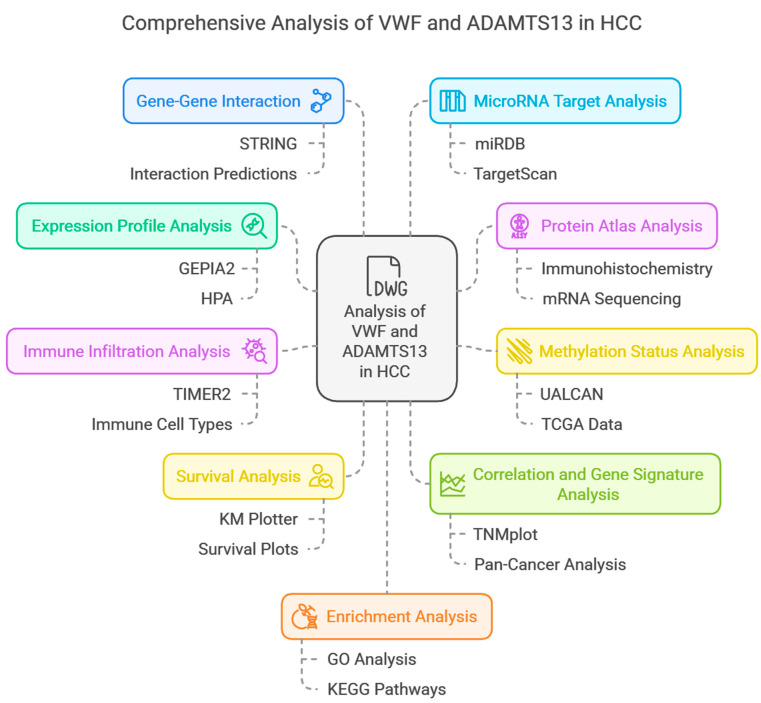
Flowchart of comprehensive analysis of *VWF* and *ADAMTS13* in HCC.

The flowchart of the current study using bioinformatics databases is shown in Figure 1.

### 2.1. The Expression Profile Analysis of VWF and ADAMTS13

The access date: 1 December 2024

URL: http://gepia2.cancer-pku.cn/

Purpose: To investigate expression profile analysis of *VWF* and *ADAMTS13* in HCC.

Process: The Gene Expression Profiling Interactive Analysis2 (GEPIA2) web database was used for expression profile analysis. GEPIA2 is an enhanced portion of the web server that offers more detailed gene expression analysis including transcription level quantification, analysis of specific cancer subtypes, and the ability of users to upload their own RNA-seq fragments to separate them from existing datasets [23]. Using the GEPIA2 web server, it was possible to compare the expression levels of genes examined in both tumoral tissues and adjacent normal tissues. It is also possible to compare the expression levels of genes examined in cancer subtypes according to the type of cancer. In our HCC cohort, we compared the expression levels of *VWF* and *ADAMTS13* genes between the adjacent normal tissue (*n* = 160) and the tumor tissue (n = 369). We also compared the expression levels of *VWF* and *ADAMTS13* genes in the iCluster1 (Proliferative/Stem Cell-Like) (n = 63), iCluster2 (Intermediate/Immune-Active) (n = 55), and iCluster3 (Non-Proliferative/Metabolic) (n = 63) subtypes of LIHC (GEPIA2; there were only 181 patients classified according to liver hepatocellular carcinoma (LIHC) subtypes with adjacent normal tissue (n = 160). We also evaluated the expression levels of *VWF* and *ADAMTS13* genes according to HCC stage (stage I, stage II, stage III, and stage IV).

### 2.2. Human Protein Atlas (HPA) Analysis

The access date: 1 December 2024

URL: https://www.proteinatlas.org/

Purpose: The Human Protein Atlas (HPA) project is a comprehensive effort focused on mapping the entire human proteome using antibody-based proteomic techniques.

Process: Tissue resource features protein expression profiles based on immunohistochemistry (IHC) for 44 normal tissues, along with mRNA expression data from 54 tissues, primarily obtained through deep mRNA sequencing. Additionally, fluorescent multiplex IHC (mIHC/IF) was used to analyze 1021 proteins in specific cell types and tissues, including ciliated cells, kidneys, testes, and salivary glands [24].

### 2.3. The Methylation Status Analysis of VWF and ADAMTS13

The access date: 1 December 2024

URL: https://ualcan.path.uab.edu/

Purpose: To investigate the promotor region methylation status of *VWF* and *ADAMTS13* in *HCC*.

Process: University of Alabama at Birmingham Cancer data analysis (UALCAN) is a comprehensive, user-friendly, interactive web resource for analyzing cancer transcriptomic data. It aims to facilitate the exploration of The Cancer Genome Atlas (TCGA) data and enable researchers to analyze gene expression profiles and perform in-depth analyses of various cancer types. UALCAN provides insights into the methylation status of gene promoters, where users can compare methylation levels between tumor and normal samples. This allowed us to study the correlation between the expression levels of these two genes. This server is useful for identifying potential gene interactions and pathways [25]. We performed promoter region methylation analyses [tumor tissue (n = 377), normal tissue (n = 50)] with the UALCAN web server and examined the expression profiles of other input genes in LIHC, including *VWF* and *ADAMTS13*.

### 2.4. Immune Infiltration Analysis of VWF and ADAMTS13

The access date: 1 December 2024

URL:  http://timer.comp-genomics.org/

Purpose: To investigate immune infiltration of *VWF* and *ADAMTS13* in HCC.

Process: TIMER2 (Tumor Immune Estimation Resource 2.0) is an advanced web server that enables comprehensive analysis of tumor-infiltrating immune cells. It builds on the functionality of its predecessor, TIMER, but provides more powerful features and an expanded dataset. TIMER2 was primarily designed to investigate the relationship between immune infiltration and various clinical and genomic features of cancer. TIMER2 provides multiple algorithms to estimate the abundance of six types of immune cells in tumor tissues: B cells, CD4+ T cells, CD8+ T cells, neutrophils, macrophages, and dendritic cells. Users can compare immune cell infiltration levels between different types of cancer and between tumor and normal tissues. TIMER2 integrates data from various sources, including The Cancer Genome Atlas (TCGA) and other large-scale cancer genomic projects. This ensured a comprehensive and up-to-date dataset for analysis [26].

### 2.5. The Survival Analysis of VWF and ADAMTS13

The access date: 1 December 2024

URL: https://kmplot.com/analysis/

Purpose: To investigate overall survival (OS) status of *VWF* and *ADAMTS13* in *HCC*.

Process: The KM Plotter (Kaplan–Meier Plotter) is an online tool designed to assess the effect of genes on survival in various cancers using clinical data. This enables researchers to conduct meta-analyses of gene expression data to identify potential prognostic biomarkers. Users can generate Kaplan–Meier survival plots to visualize the relationship between gene expression levels and patient survival outcomes. It integrates data from several high-quality sources such as the Gene Expression Omnibus (GEO), European Genome-phenome Archive (EGA), and TCGA and combines clinical data with gene expression profiles to provide a comprehensive analysis [27].

### 2.6. The Correlation, Targetgram, and Gene Signature Analysis of VWF and ADAMTS13

The access date: 1 December 2024

URL: https://tnmplot.com/analysis/

Purpose: To investigate the correlation, Targetgram, and gene signature of VWF and ADAMTS13 in HCC.

Process: The TNMplot database allows users to compare gene expression changes in real-time across tumor, normal, and metastatic tissues for all genes using various platforms. The analysis portal is accessible without registration at its original website (www.tnmplot.com accessed on 1 December 2024) and offers three distinct analysis options. One of these, the pan-cancer analysis tool, enables the simultaneous comparison of normal and tumor samples across 22 different tissue types [28].

### 2.7. The Gene–Gene Interaction

The access date: 1 December 2024

URL: https://string-db.org/

Process: We used the STRING database to reveal the interactions and possible mechanisms of VWF and ADAMTS13 with other related proteins. The STRING database systematically predicts protein–protein interactions including both physical and functional associations. Data were provided from sources such as automated text mining of the scientific literature, computational interaction predictions from co-expression, conserved genomic context, and databases of interaction experiments. All these interactions are critically evaluated and scored and then automatically transferred to less-studied organisms using hierarchical orthology information [29].

### 2.8. MicroRNA Target Analysis

The access date: 1 December 2024

URL: https://mirdb.org/

https://www.targetscan.org/vert_80/

Purpose: To investigate target miRNAs of *VWF* and *ADAMTS13* in HCC. By identifying miRNAs associated with these genes, the study aimed to uncover regulatory networks that could provide further insights into their roles in HCC pathogenesis and progression.

Process: We used the MicroRNA Target Prediction Database (miRDB) [30] and TargetScan 8.0 to identify and predict the target genes of differentially expressed miRNAs [31,32].

### 2.9. Enrichment Analysis of ADAMTS13 and VWF (Enrichr-KG)

The access date: 1 December 2024

URL: https://maayanlab.cloud/enrichr-kg

Purpose: To investigate gene enrichment of ADAMTS13 and VWF.

Process: Differentially expressed gene (DEG) analysis was conducted using the Enrichr-KG web tool across several categories: Gene Ontology (GO) biological process (https://geneontology.org/ accessed on 1 December 2024), Kyoto Encyclopedia of Genes and Genomes (KEGG) (https://www.genome.jp/kegg/ accessed on 1 December 2024), Jensen_DISEASES (https://diseases.jensenlab.org/Search accessed on 1 December 2024) for exploring disease–gene associations, and DisGeNET (https://disgenet.com/) for compiling data on genes and variants linked to diseases. The analysis was limited to the top 20 terms, with statistical significance defined as *p* < 0.05 [33].

### 2.10. Association of HCC-Associated Long Non-Coding RNAs (LncRNAs) with VWF and ADAMTS13

The access date: 20 January 2025

URL: http://www.cuilab.cn/lncrnadisease

Purpose: To predict HCC-associated long non-coding RNAs.

Process: We used the LncRNADisease database to find long non-coding RNAs associated with HCC. The lncRNADisease database serves as both a repository of experimentally validated lncRNA–disease association data and a platform offering tools to predict potential novel lncRNA–disease associations. Furthermore, it compiles data on lncRNA interactions across multiple levels including proteins, RNAs, miRNAs, and DNA [34].

URL: https://rnasysu.com/encori/

Purpose: To investigate the association of LncRNAs with VWF and ADAMTS13.

Process: We used the ENCORI database to examine the relationship between long non-coding RNAs with *VWF* and *ADAMTS13*. The ENCORI Pan-Cancer Analysis Platform was developed to unravel pan-cancer networks involving lncRNAs, miRNAs, pseudogenes, snoRNAs, RNA-binding proteins (RBPs), and all protein-coding genes. This was achieved by analyzing the expression profiles from approximately 10,000 RNA-seq and 9900 miRNA-seq samples across 32 cancer types, integrated from TCGA project [35].

### 2.11. Detection of Differentially Expressed Genes (DEGs)

The access date: 25 February 2025

URL: https://www.ncbi.nlm.nih.gov/gds

Purpose: To evaluate differential gene expression analysis of VWF and ADAMTS13.

Process: The Gene Expression Omnibus (GEO) datasets were used in this study. The datasets analyzed were GSE14520 [36,37,38,39,40,41,42,43,44,45,46,47,48] using the GPL10558 [HCC (n = 222), control (n = 212)] platform Affymetrix HT Human Genome U133A Array, Bethesda, MD, USA.

For gene expression profiling, tumors and paired non-tumor tissues were profiled separately using a single channel array platform. Tumor and paired non-tumor samples of 22 patients of cohort1 and the normal liver pool were carried out on Affymetrix GeneChip HG-U133A 2.0 arrays (Affymetrix, Santa Clara, CA, USA) according to the manufacturer’s protocol.

Analyses were conducted using the GEO2R tool (https://www.ncbi.nlm.nih.gov/geo/geo2r/ accessed on 25 February 2025) to identify differentially expressed genes (DEGs) in both datasets. This tool operates using GEOquery and limma to process microarray data and detect DEGs. In this study, multiple testing corrections were applied using the Benjamini and Hochberg false discovery rate method to compute the adjusted *p*-values. A log2 fold change threshold of 1 was used, and the significance level for the adjusted *p*-value was maintained at 0.05 by default. Genes with an adjusted *p*-value below 0.05 and Log2(FC) < −1 were classified as downregulated, while those with an adjusted *p*-value below 0.05 and Log2(FC) > 1 were identified as upregulated [49].

### 2.12. Statistical Analysis Methods

*VWF* and *ADAMTS13* expression profiles were compared between HCC tumors and adjacent normal tissues. A *t*-test was used to determine statistical significance. The protein expression levels were examined using IHC and mRNA expression data. This was supported by visual analysis. Promoter methylation status was compared between tumor and normal tissues. Differences between methylation levels were analyzed using the Mann–Whitney U test. Immune cell infiltration of tumor tissue was analyzed. The relationships between *VWF* and *ADAMTS13* expression and immune cell subgroups were calculated using the Pearson correlation coefficient. The Kaplan–Meier method was used for survival analyses. The effects of *VWF* and *ADAMTS13* expression on OS were evaluated using the log-rank test. Changes in *VWF* and *ADAMTS13* expression were compared among tumor, normal, and metastatic tissues. Statistical significance was determined using the Mann–Whitney U test. Gene–gene interaction networks were constructed, and functional relationships were evaluated by combined scores. The Spearman correlation coefficients were used to assess correlations among miRNA, lncRNA, and other genes associated with *VWF* and *ADAMTS13*.

## 3. Results

### 3.1. Expression Profile of VWF and ADAMTS13

**Figure 2:**
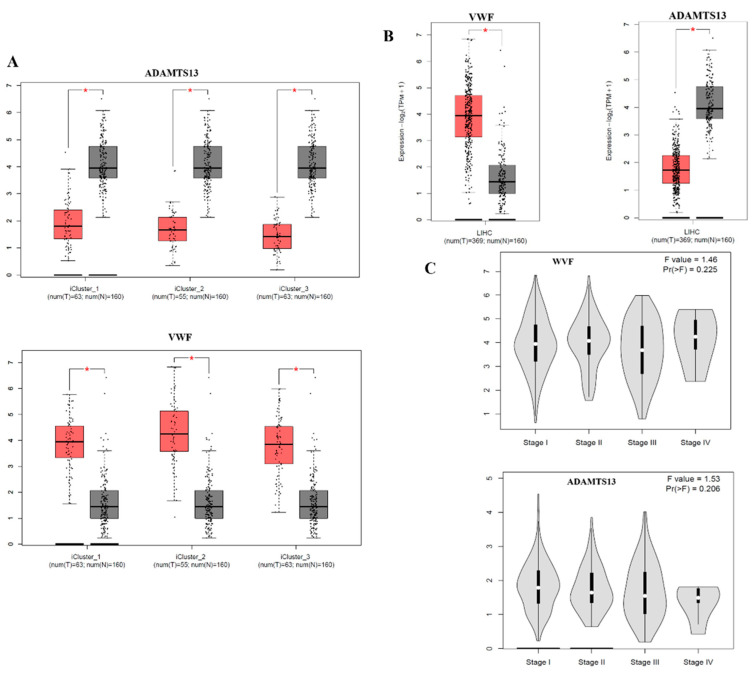
(**A**) The *VWF* and *ADAMTS13* expression results in LIHC subtypes are demonstrated using the GEPIA2 webtool. (**B**) *VWF* and *ADAMTS13* expression results in LIHC are shown in the GEPIA2 webtool. (**C**) Changes in *VWF* and *ADAMTS13* expression according to LIHC stage. * *p* < 0.05 is statistically significant.

According to the subtype analysis of LIHC, the *VWF* expression level was significantly upregulated in all subtypes of LIHC compared to the adjacent normal tissue. In contrast, the expression level of *ADAMTS13* was statistically downregulated in all subtypes of LIHC compared to adjacent normal tissue (Figure 2A).

When the expression profiles of *VWF* and *ADAMTS13* in LIHC patients were investigated, the VWF expression level was significantly upregulated in LIHC tissue (n = 369) compared to that in adjacent normal tissue (n = 160) (*p* < 0.05). In contrast, ADAMTS13 expression was significantly downregulated in LIHC tissues (n = 369) compared to adjacent normal tissues (n = 160) (*p* < 0.05) (Figure 2B).

Neither *VWF* nor *ADAMTS13* expression levels were statistically significant at the LIHC stage (*p* > 0.05) (Figure 2C).

**Figure 3:**
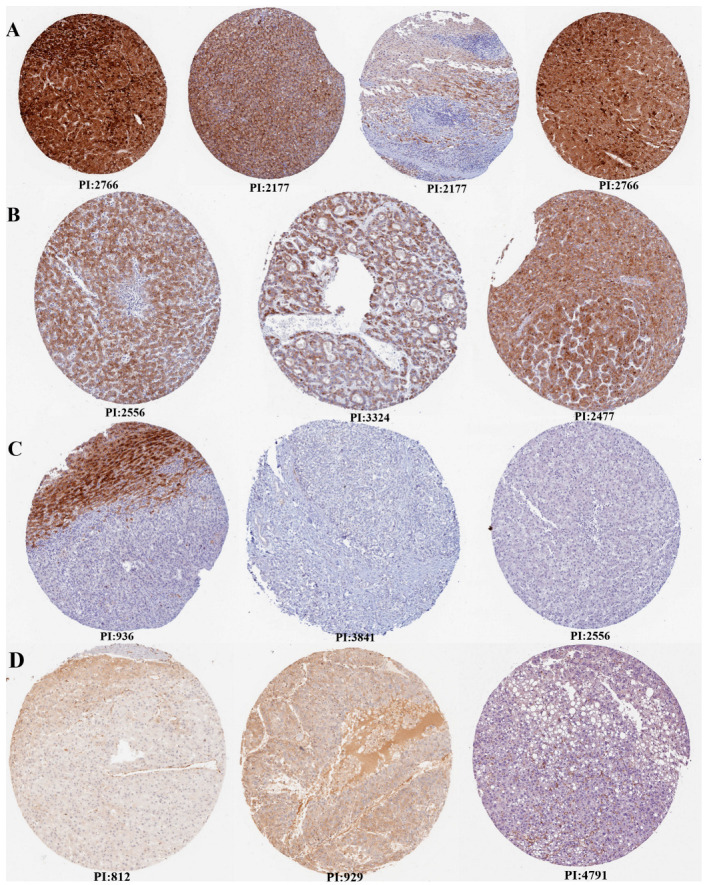
Expression verification of *ADAMTS13* and *VWF* by Human Protein Atlas (HPA). (**A**) High tumor staining for ADAMTS13, (**B**) medium tumor staining for ADAMTS13, (**C**) not detected tumor staining for ADAMTS13, (**D**) not detected tumor staining for VWF (there is no high, medium, or low level tumor staining in HPA for VWF). Scale bar = 200 µm.

Additionally, the expression verification of *VWF* and *ADAMTS13* was performed using HPA (Figure 3). Accordingly, there was high, medium, and low tumor staining in LIHC tumor tissues for *ADAMTS13.* However, this was not the case for *VWF*.

### 3.2. Promoter Methylation Status of VWF and ADAMTS13

**Figure 4:**
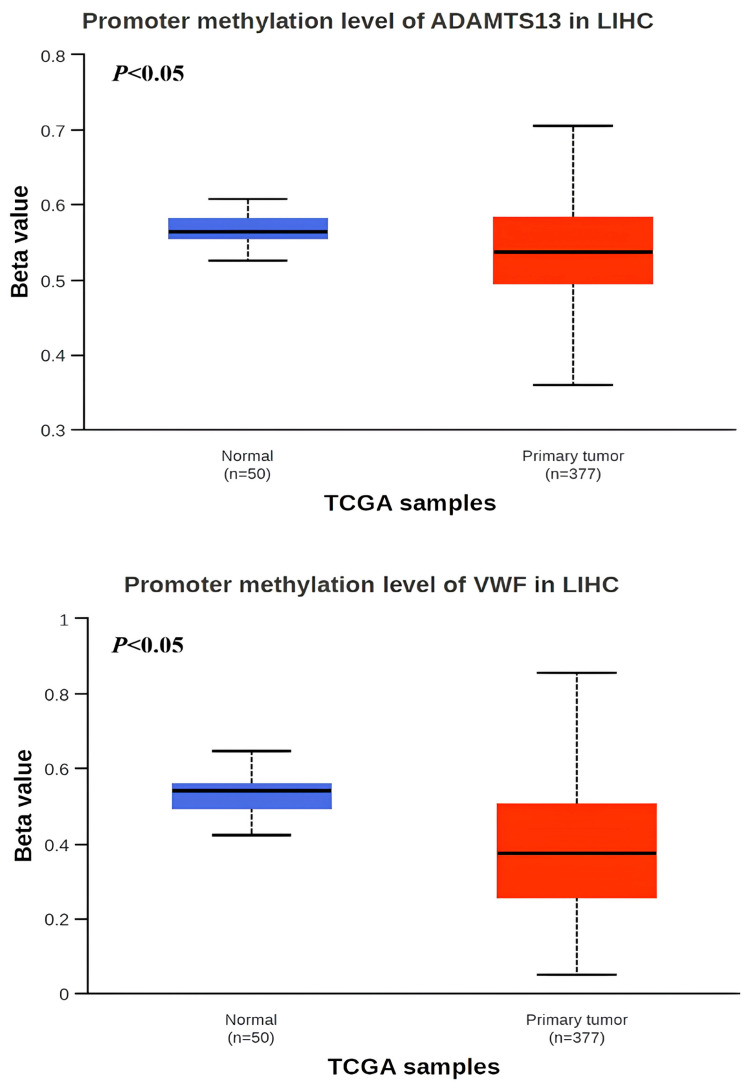
Promotor region methylation of *VWF* and *ADAMTS13* in LIHC.

**Figure 5:**
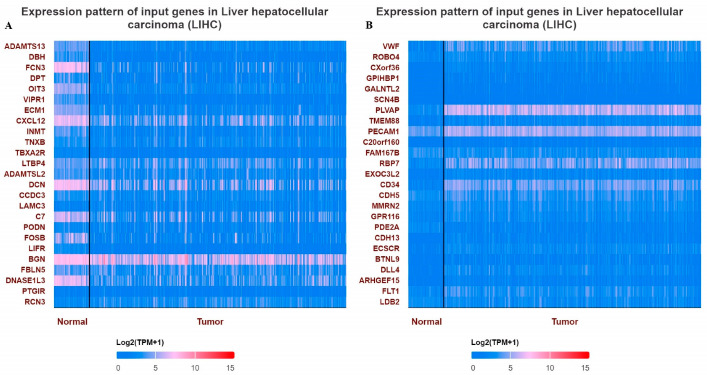
Heatmaps showing top differentially expressed genes in LIHC. (**A**) Expression pattern of top 25 input genes with VWF in LIHC, (**B**) expression pattern of top 25 input genes with *ADAMTS13* in LIHC.

When the methylation status of *VWF* and *ADAMTS13* in LIHC patients was investigated, the *VWF* methylation level was significantly hypomethylated in LIHC tissue (n = 377) compared to adjacent normal tissue (n = 50) (*p* < 0.05). Similarly, the *ADAMTS13* methylation level was significantly hypomethylated in LIHC tissue (n = 377) compared to adjacent normal tissue (n = 50) (Figure 4). In addition, the expression pattern of the input genes in LIHC is shown in Figure 5A,B.

### 3.3. Survival Analysis Results of *VWF* and *ADAMTS13*

**Figure 6:**
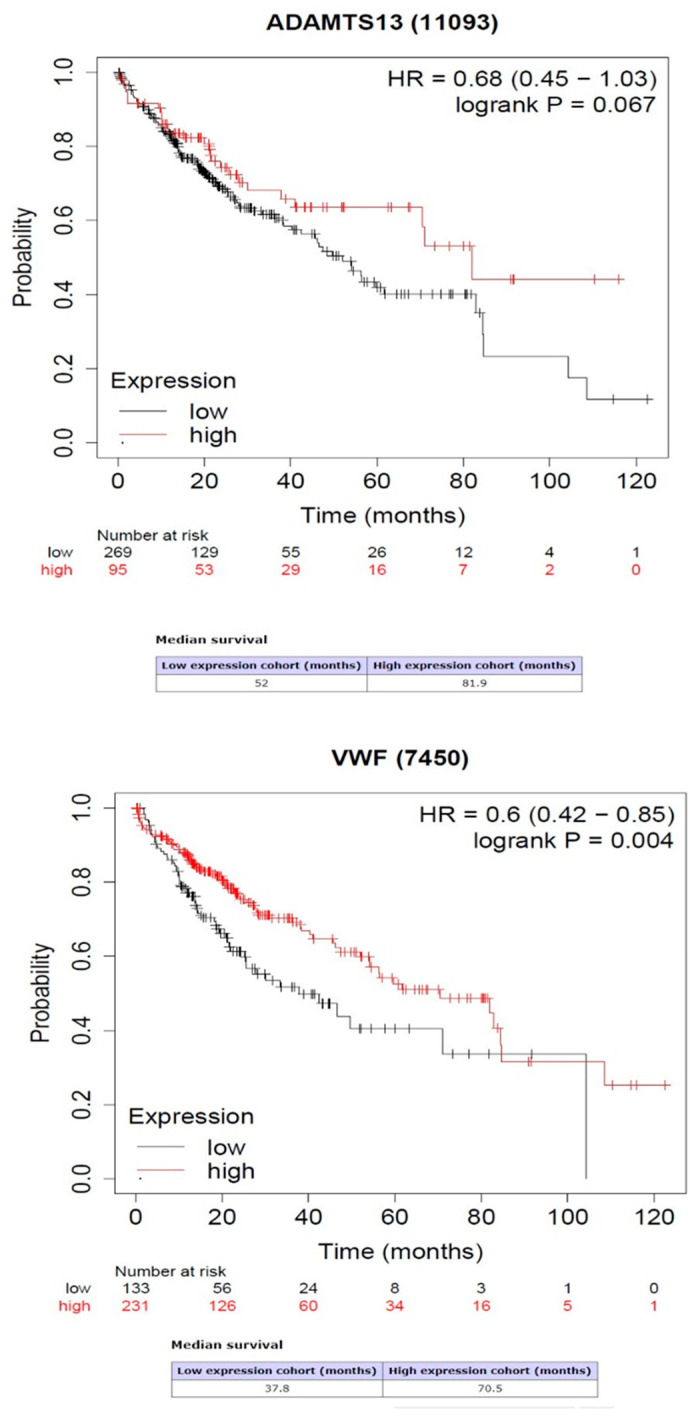
Association between gene expression of *ADAMTS13* and *VWF* with overall survival.

Kaplan–Meier survival analysis showed that the high expression of *VWF* predicted high rates of overall survival (OS) (*p* = 0.004). However, the high or low expression of *ADAMTS13* was not related to OS prediction (*p* = 0.064) (Figure 6).

### 3.4. Immune Infiltrates and Gene Expression of ADAMTS13 and VWF

**Figure 7:**
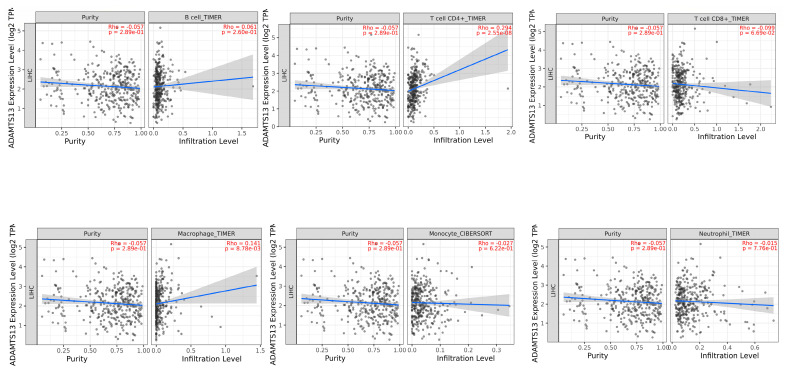
Association of tumor-infiltrating immune cells (TIICs) with *ADAMTS13* expression.

A significant positive correlation was observed between ADAMTS13 with CD4+ T cells (r = 0.294, *p* = 2.55 × 10^−8^) and macrophages (r = 0.141, *p* = 8.78 × 10^−3^). (Figure 7).

**Figure 8:**
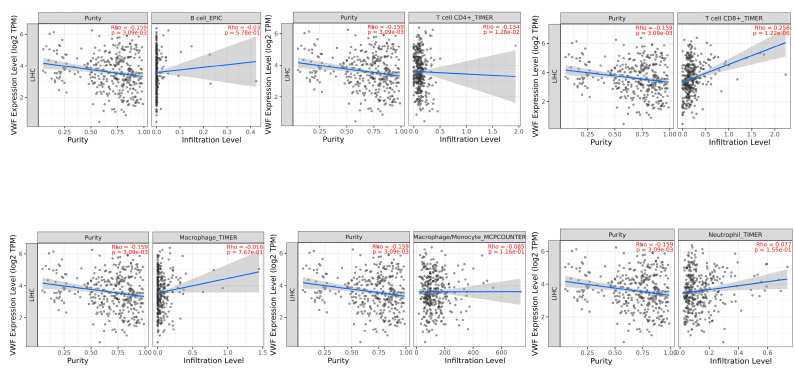
Association of tumor-infiltrating immune cells (TIICs) with *VWF* expression in LIHC.

TIMER2 immune association analysis showed a significant negative correlation between VWF and tumor purity (proportion of tumor cells in the tumor microenvironment) (r = −0.159, *p* = 3.09 × 10^−3^) and CD4 + T cells (r = −0.134, *p* = 1.28 × 10^−2^). In contrast, a significant positive correlation was observed between VWF and CD8 + T cells (r = 0.258, *p* = 1.22 × 10^−6^), whereas there was a significant negative correlation between VWF and tumor purity (r = −0.159, *p* = 3.09 × 10^−3^) (Figure 8).

### 3.5. TNMplot Analysis of VWF and ADAMTS13

**Figure 9:**
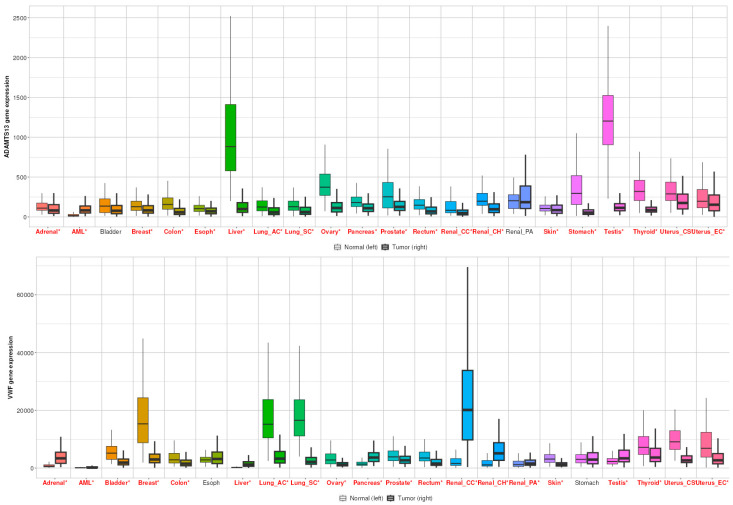
Differential gene expression results of *VWF* and *ADAMTS13* in all cancer types. Red ones represent statistically significant level (*p* < 0.05).

When all cancer types were examined, *ADAMTS13* expression was significantly altered in all cancer types, except bladder and renal PA (Figure 9). In contrast, *VWF* expression was significantly altered in other cancer types, except esophageal and gastric cancer (Figure 9).

**Figure 10:**
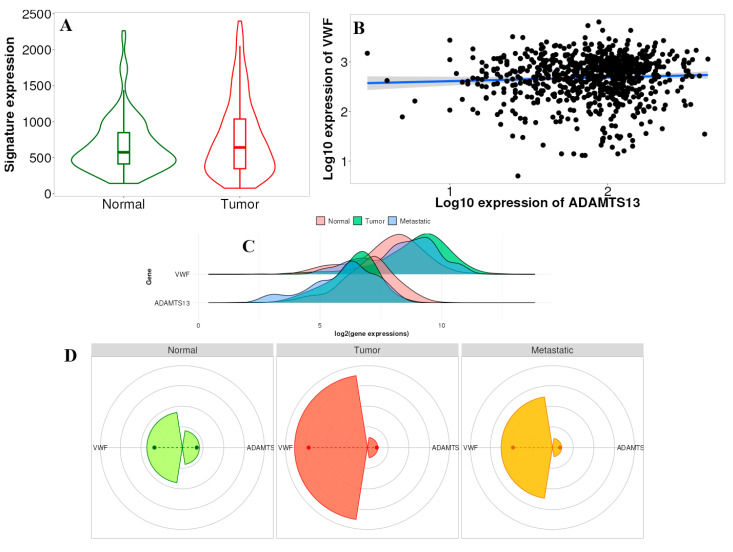
(**A**) Gene signature result of ADAMTS13 and VWF in normal tissue vs. tumor tissue (*p* < 0.05). (**B**) Comparison of correlation between VWF and ADAMTS13 using multiple correlation methods in selected tissue type based on RNA-Seq data (r = −0.15, *p* = 0.02, Spearman correlation). (**C**) Multigene analysis showed expressions of VWF and ADAMTS13 in normal, tumor, and metastatic tissues. (**D**) Targetgram analysis of VWF and ADAMTS13 in normal, tumor, and metastatic tissues.

According to the results, the difference in gene signature analysis between tumor and normal tissues was statistically different in the direction of upregulation in the tumor tissue (*p* = 0.024) (Figure 10A). In addition, according to the multigene analysis results, there was an inverse relationship between *VWF* and *ADAMTS13* expression in LIHC tissues (Figure 10B). Changes in the expression of *ADAMTS13* and *VWF* in normal, tumor, and metastatic tissues are shown in Figure 10C. Gene signature analysis calculates the averages of the selected gene signatures for each patient and presents a summary graph using RNA-Seq-based data. The Targetgram page provides an overview of the selected gene set in the selected tissue using gene chip-based data; the size of the segments represents the mean values, and the length of the dashed lines represents the median values of each type. According to the results of this analysis, the median VWF was the highest in tumor tissue (Figure 10D).

### 3.6. Gene–Gene Interaction Combined Score Results

**Figure 11:**
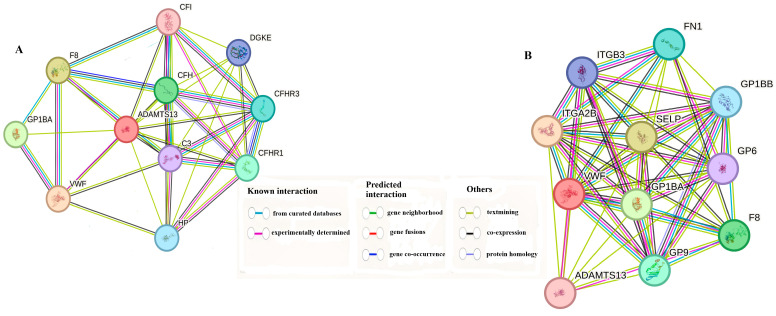
(**A**) Genes associated with *ADAMTS13*. (**B**) Genes associated with *VWF* gene.

The 10 genes that interacted most with both *VWF* and *ADAMTS13* are shown in Table 1 according to their combined scores. In addition, a schematic representation of the interactions of the same genes is given in Figure 11A,B.

### 3.7. MicroRNA Target Analysis Result

**Figure 12:**
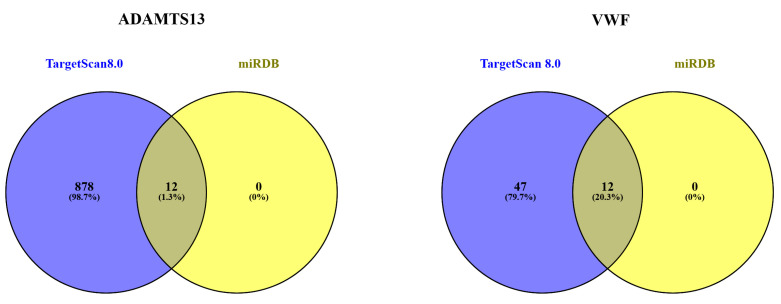
Separate and overlapping numbers of miRNAs that are potential targets of *ADAMTS13* and *VWF* according to miRDB and TargetScanHuman8.0.

The miRNAs associated with *ADAMTS13* and *VWF* are listed in Table 2. Accordingly, the miRNAs were grouped as a combination of TargetScan Human 8.0 and miRDB databases (Figure 12)**.**

### 3.8. Enrichment Analysis of ADAMTS13 and VWF

#### 3.8.1. Association of ADAMTS13 with Physiopathological Processes

According to Gene Ontology, ADAMTS13 is involved in the catabolic process of organonitrogen compounds (GO:1901565), proteolysis (GO:0006508), protein maturation (GO:0051604), protein processing (GO:0016485), extracellular matrix organization (GO:0030198), glycoprotein metabolic process (GO:0009100), extracellular structure organization (GO:0043062), peptide metabolic process (GO:0006518), cell–matrix adhesion (GO:0007160), integrin-mediated signaling pathway (GO:0007229), external encapsulating structure organization (GO:0045229), and peptide catabolic process (GO:0043171).

Data from FANTOM6 shows that the knockdown of certain lncRNAs can regulate ADAMTS13 expression. The lncRNAs AC017048.3-ASO_G0163364_AD_01-DEGs, RP11-458D21.1-ASO_G0233396_07-DEGs, MEG3-ASO_G0214548_AD_09-DEGs, and ERVK3-1-ASO_G0142396_AD_07-DEGs upregulated ADAMTS13 expression, whereas the lncRNAs RAB30-AS1-ASO_G0246067_AD_06-DEGs, CD27-AS1-ASO_G0215039_01-DEGs, RP11-473M20.14-ASO_G0263072_06-DEGs, and RP11-395B7.4-ASO_G0227053_04-DEGs downregulated its expression.

Jensen Lab data links ADAMTS13 to several diseases, including thrombotic thrombocytopenic purpura, thrombocytopenia, Weill–Marchesani syndrome, hypertension, cerebrovascular disease, carcinoma, and diarrhea.

**Figure 13:**
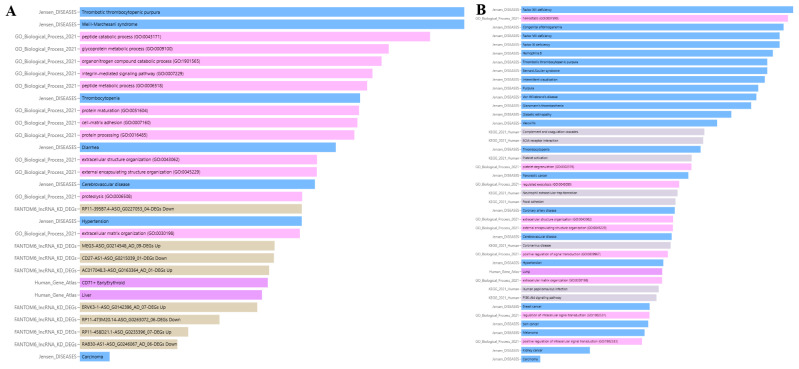
Enrichment analysis results of *ADAMTS13* (**A**) and *VWF* (**B**).

According to the Human Gene Atlas, ADAMTS13 is upregulated in CD71+ early erythroid and liver cells, as shown in Figure 13A and Table 3.

#### 3.8.2. Association of VWF with Physiopathological Processes

Gene Ontology analysis indicates that VWF is involved in the following biological processes: regulated exocytosis (GO:0045055), positive regulation of signal transduction (GO:0009967), hemostasis (GO:0007599), extracellular structure organization (GO:0043062), regulation of intracellular signal transduction (GO:1902531), platelet degranulation (GO:0002576), positive regulation of intracellular signal transduction (GO:1902533), extracellular matrix organization (GO:0030198), and external encapsulating structure organization (GO:0045229).

According to KEGG pathway analysis, VWF is involved in the following pathways: neutrophil extracellular trap formation, ECM-receptor interaction, complement and coagulation cascades, human papillomavirus infection, focal adhesion, PI3K-Akt signaling pathway, coronavirus disease, and platelet activation.

Jensen Lab data associates VWF with several diseases, including pancreatic cancer, thrombotic thrombocytopenic purpura, hemophilia B, intermittent claudication, Von Willebrand’s disease, purpura, coronary artery disease, diabetic retinopathy, Factor VIII deficiency, thrombocytopenia, kidney cancer, breast cancer, Bernard–Soulier syndrome, vasculitis, Factor XIII deficiency, hypertension, Glanzmann’s thrombasthenia, cerebrovascular disease, carcinoma, melanoma, skin cancer, congenital afibrinogenemia, and Factor XI deficiency.

The Human Gene Atlas shows that *VWF* is upregulated in lung cells, as shown in Figure 13B and Table 4.

#### 3.8.3. LncRNAs Associated with HCC and Their Association with VWF and ADAMST13

**Figure 14:**
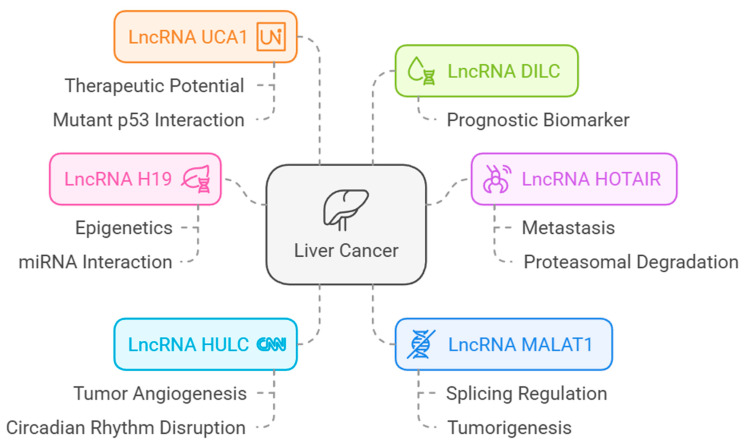
LncRNAs associated with HCC [from LncRNADisease database (http://www.cuilab.cn/lncrnadisease accessed on 25 February 2025)].

The lncRNAs associated with HCC are shown in Figure 14.

#### 3.8.4. Correlation of lncRNAs with ADAMTS13 and VWF

**Figure 15:**
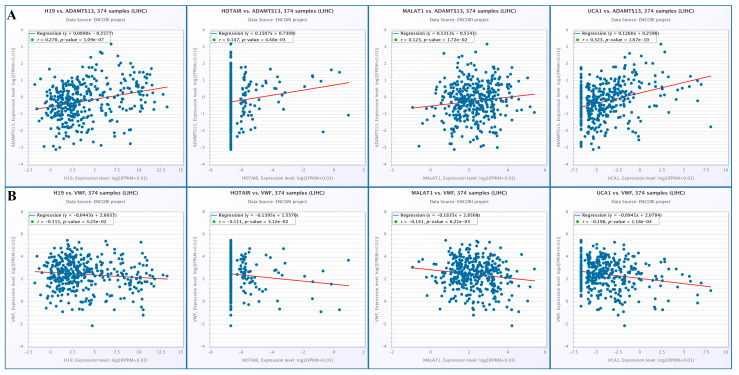
(**A**) Changes in lncRNA and ADAMTS13 expression. (**B**) Changes in LncRNAs expressions and *VWF* expressions (from Encori database, no data were found in Encori database for HULC and DILC).

When the changes in lncRNA expression with *VWF* and *ADAMTS13* expression were examined, there was a positive correlation among *ADAMTS13* expression and H19, HOTAIR, MALAT1, and UCA1 expression (r = 0.270, *p* = 1.09 × 10^−7^, r = 0.147, *p* = 4.48 × 10^−3^, r = 0.123, *p* = 1.72 × 10^−2^, r = 0.323, *p* = 1.67 × 10^−10^, respectively) (Figure 15A). In contrast, there was a negative correlation among *VWF* expression and H19, HOTAIR, MALAT1, and UCA1 expression (r = −0.111, *p* = 3.25 × 10^−2^, r = −0.111, *p* = 3.12 × 10^−2^, r = −0.141, *p* = 6.21 × 10^−3^, r = −0.198, *p* = 1.18 × 10^−4^, respectively) (Figure 15B).

### 3.9. Analysis Results of DEGs

**Figure 16:**
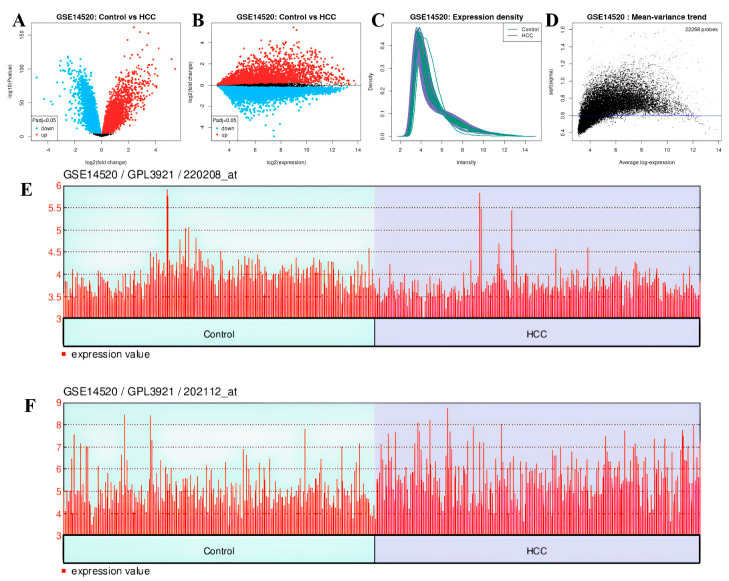
In GSE14520 dataset: (**A**) volcano plot and (**B**) mean difference plot views of data distribution, (**C**) expression density, (**D**) mean variance trend, (**E**) expression value plot of *VWF* (control vs. HCC), (**F**) expression value plot of *ADAMTS13* (control vs. HCC).

In the GSE14520 dataset, 12.298 genes were upregulated and 9.970 genes were downregulated (Figure 16A,B). The expression density and mean variance trend of the GSE14220 dataset are shown in Figure 16C,D.

The downregulated genes were VWF (adjp = 3.87 × 10^−7^, logFC = −0.606) with gene ID: 202112 (Figure 16E) and ADAMTS13 (adjp = 1.01 × 10^−6^, log = 0.214) with gene ID: 220208 (Figure 16F). Genes showing separate and common upregulation in the datasets are listed in Supplementary Table S1.

## 4. Discussion

Our current study is the first to compare the role of VWF and ADAMTS13 in HCC using different bioinformatic databases and microarray data. To our knowledge, there is no such large-scale study in the literature. Based on the correlations we have made and the data we have gathered, we believe that our study will enable the further elucidation of such physiopathological associations and serve as an impetus for further research.

VWF is a large multimeric glycoprotein that is synthesized by endothelial cells and megakaryocytes. Its main function is to ensure the binding of platelets to areas of vascular damage in the first stage of homeostasis [50]. ADAMTS13 is a metalloproteinase that cleaves large multimers of VWF and thus regulates the hemostatic activity of VWF. Increased VWF levels and decreased ADAMTS13 activity have also been observed in HCC. This imbalance is associated with tumor angiogenesis and metastasis [20]. VWF may facilitate tumor cell adhesion to the endothelium and subsequent metastasis [20]. At the same time, low levels of ADAMTS13 may enhance the pro-tumor effects of VWF. Several studies have suggested that VWF and ADAMTS13 levels may have prognostic value in patients with HCC. For example, high *VWF* levels and low *ADAMTS13* activity have been associated with poorer prognosis and shorter survival times in the advanced stages of the disease. These biomarkers could potentially be used to monitor patients with HCC and to determine treatment strategies [8,15]. While the effects of *VWF* and *ADAMTS13* expression levels on prognosis in advanced-stage HCC are in this direction, VWF was upregulated, while *ADAMTS13* levels were downregulated in our HCC cohort. However, when the markers were evaluated in terms of OS, it was observed that ADAMTS13 gene expression levels had no effect on OS, whereas increasing VWF expression levels were associated with longer OS. Elevated VWF expression, identified as a positive prognostic marker, could be developed into a routine biomarker to stratify patients with HCC based on their survival probabilities. This stratification can help identify patients who may benefit from more aggressive therapies or closer monitoring. The *VWF* and *ADAMTS13* pathways can be explored as potential therapeutic targets. Modulating *VWF* expression may enhance tumor suppression, and addressing the imbalance between *VWF* and *ADAMTS13* may reduce angiogenesis and metastasis. Contrary to previous studies, we can say that only high VWF expression levels were a positive prognostic factor for patients in our HCC cohort. This may be because the cohort we examined included patients from all stages, not only advanced patients.

Other mechanisms may explain the upregulation of VWF gene expression levels. The first is hypomethylation of the promoter region, which is an epigenetic mechanism. Hypomethylation was observed in the VWF promoter region of our HCC cohort. This hypomethylation may have resulted in the upregulation of the VWF gene. A recent study showed that the overexpression of *VWF* in breast cancer cells leads to an increase in VEGF-A-related angiogenesis [51]. Second, evidence suggests that in certain cancer patients, *VWF* is released not only from typical sources, such as endothelial cells and megakaryocytes, but also from tumor cells and the surrounding tumor microenvironment.

The contradiction between hypomethylation and the downregulation of *ADAMTS13* gene levels in our HCC cohort can be explained by global DNA methylation. Global DNA hypomethylation in HCC may be associated with the formation of repressive chromatin domains and gene silencing. Global DNA hypomethylation, which is frequently observed in cancer, is believed to activate potential oncogenes and cause chromosomal changes, thereby playing a role in carcinogenesis. Chromosomal abnormalities suggest that global hypomethylation may significantly contribute to chromosomal instability [52]. In addition to this, miRNAs or RNA-binding proteins may inhibit the translation of ADAMTS13, even in the presence of hypomethylation. Hypomethylation alone does not ensure transcriptional activation, as specific transcription factors must be both present and active. Furthermore, histone modifications or chromatin remodeling may counteract the effects of DNA hypomethylation, thereby restricting gene expression. Additionally, cytokines, inflammatory signals, or hypoxia within the tumor microenvironment may regulate ADAMTS13 levels independently of methylation status. However, the relationship between these features during the development of HCC remains uncertain, owing to the HCC having multiple reasons and mechanisms. The downregulation of the *ADAMTS13* gene versus promoter region hypermethylation may be only one of the epigenetic reasons or mechanisms contributing to the pathogenesis of HCC.

In addition, in the analysis of DEGs using microarray data, we obtained results that were exactly the opposite of those obtained in other bioinformatic databases. While VWF was downregulated in the DEGs analysis, ADAMTS13 levels were upregulated. These results may be due to working in different populations. Different results obtained from different databases (TCGA database and microarray data) regarding VWF and ADAMTS13 draw attention to the imbalance between VWF:Ag/ADAMTS13:AC in HCC. There may be many reasons for this imbalance (such as methylation profile change, effects of miRNAs and lncRNAs). Therefore, the mechanisms need to be evaluated carefully. This reason may be due to HAIC applied in the treatment of HCC. It was also shown that HAIC treatment reduces VEGF levels in patients with advanced HCC [8,53]. Moreover, the VWF:Ag/ADAMTS13:AC ratio was reported to be lower in HCC patients treated with HAIC. Therefore, the expression levels of low *VWF* and high *ADAMTS13* in the GEO dataset may be linked to the response to HAIC via their effects on VEGF and angiogenesis.

The tumor microenvironment (TME) is integral to the development of hepatocellular carcinoma (HCC), influencing key processes such as cell proliferation, migration, invasion, epithelial–mesenchymal transition (EMT), immune evasion, angiogenesis, and treatment resistance [54]. Critical components of the HCC microenvironment, including blood vessels, hepatic non-parenchymal cells (e.g., Kupffer cells, hepatic stellate cells, and liver sinusoidal endothelial cells), and various lymphocyte populations, significantly affect tumor initiation and progression [55]. Among these, CD8+ T cells are pivotal for tumor control due to their capacity to recognize cancer-specific antigens and exert cytotoxic effects through the release of perforin and granzyme B [56]. Although the increased infiltration of CD8+ T cells is generally associated with improved survival in many cancers, its prognostic significance in HCC remains contentious [57,58]. In the present study, elevated VWF expression was correlated with longer overall survival (OS) in HCC patients and showed a significant positive association with CD8+ T cell levels. Conversely, VWF expression demonstrated a negative correlation with CD4+ T cells. CD4+ T lymphocytes play a dual role in cancer by activating cytotoxic CD8+ T cells through cytokine release, but under certain conditions, they may also impair CD8+ T cell functionality, contributing to disease progression in HBV-related HCC [58]. This imbalance between CD4+ and CD8+ T cells may accelerate tumor progression and serve as a valuable prognostic indicator [59,60]. Notably, patients with HBV-related HCC exhibit higher CD4+ T cell levels than those with chronic hepatitis or healthy individuals, although these levels decline in advanced stages, particularly within the tumor core and peritumoral regions, potentially due to selective recruitment aimed at immune evasion [61]. Reduced tumor-infiltrating CD4+ T lymphocytes (CD4+ TILs) are associated with poor prognosis in HCC [61]. Furthermore, findings in the HCC cohort demonstrated an inverse relationship between VWF and CD4+ T lymphocytes, while ADAMTS13 expression showed a positive correlation with CD4+ T cells. Given the observed downregulation of ADAMTS13 and the upregulation of VWF, the expected immune profile includes decreased CD4+ T cell levels and increased CD8+ T cell levels. This suggests that CD4+ TILs are not only reduced in number but also functionally impaired within the tumor microenvironment. Over time, this deterioration may lead to compromised activation of CD8+ TILs in advanced disease stages, potentially contributing to a poorer prognosis in HCC. The upregulation of VWF in HCC appears to serve as a negative prognostic indicator, as it is correlated with a reduction in CD4+ TILs and a potential impairment in the activation of CD8+ TILs. This phenomenon may facilitate immune evasion and contribute to poorer long-term outcomes in HCC.

Enrichment analysis of *ADAMTS13* and *VWF* adds significant value to the discussion by providing deeper insights into their roles in HCC. It links these genes to critical cancer-related processes such as angiogenesis, extracellular matrix organization, and immune regulation, explaining how their dysregulation may influence tumor progression. Additionally, the identification of key pathways, including PI3K-Akt signaling and ECM-receptor interactions, which are critical for tumor growth, metastasis, and immune evasion, highlights mechanisms that could be targeted therapeutically [62]. Our enrichment analysis findings indicate that VWF and ADAMTS13 are integral to coagulation, extracellular matrix organization, and disease pathogenesis. The involvement of VWF in various cancers and cardiovascular diseases underscores its potential as a biomarker or therapeutic target. Similarly, the regulation of ADAMTS13 by lncRNAs presents new opportunities for exploring gene regulation mechanisms in coagulation disorders. Further research could investigate whether analogous regulatory mechanisms are applicable to other coagulation-related factors and metalloproteinases. This integration may ensure that the enrichment analysis aligns with the study objectives and may enhance the overall understanding of *ADAMTS13* and *VWF* in HCC.

These lncRNAs play significant roles in cancer progression by promoting angiogenesis, epithelial–mesenchymal transition (EMT), and immune evasion [63]. In the cohort we examined, ADAMTS13 was downregulated, while VWF was upregulated. In addition, H19, HOTAIR, MALAT1, and UCA1 expression levels showed a negative correlation with ADAMTS13 expression levels, while they showed a positive correlation with VWF expression levels. According to these results, considering the relationship between both ADAMTS13 and VWF and lncRNAs, we expect H19, HOTAIR, MALAT1, and UCA1 to be downregulated. Downregulation of these lncRNAs (H19, HOTAIR, MALAT1, and UCA1) may lead to an imbalance between ADAMTS13 and VWF. Studies suggest that the imbalance between ADAMTS13 and VWF is associated with angiogenesis and hypercoagulability and that this is important for cancer prognosis [10,20]. It is reported in the literature that increased levels of H19, HOTAIR, MALAT1, and UCA1, which have oncogenic characteristics, are associated with HCC [64]. A study reported that MALAT1 can be downregulated through autophagy inhibition, which suppresses cell proliferation and tumor growth in HCC [65]. Another study reported that MALAT1 upregulation promoted HCC cell proliferation, while MALAT1 downregulation promoted HCC apoptosis and autophagy [66].

Investigations concerning UCA1 and HOTAIR, analogous to those on MALAT1, have primarily concentrated on the downregulation of these lncRNAs and the subsequent diminution in cancer progression [67,68]. Consequently, while investigations are ongoing regarding the potential of these three lncRNAs as therapeutic targets, the function of H19 in HCC remains complex and subject to debate. Some studies have suggested that H19 acts as an oncogene, promoting cell proliferation, migration, and invasion. Other studies have indicated that H19 may have tumor suppressor functions in HCC [69,70,71]. Cancer is a disease with a very complex nature, and angiogenesis has a very important role in cancer progression [72]. From this perspective, we may consider that H19, HOTAIR, MALAT1, and UCA1 may be downregulated to support angiogenesis by creating an imbalance between ADAMTS13 and VWF, but these need to be supported by further studies. These findings underscore the complexity of the regulatory networks involving lncRNAs, VWF, and ADAMTS13. Future studies should explore the functional roles of these lncRNAs in modulating the expression or activity of VWF and ADAMTS13. Experimental validation using knockdown or overexpression models of these lncRNAs could elucidate their direct impact on tumor behavior and clarify their contribution to HCC pathogenesis. This analysis may provide a foundation for the identification of novel therapeutic targets. Targeting specific lncRNAs, such as H19 or HOTAIR, may indirectly influence VWF and ADAMTS13 expression, thereby offering new strategies for intervention in HCC. Moreover, integrating these insights with the existing knowledge of the tumor microenvironment and epigenetic regulation could enhance our understanding of HCC progression and improve therapeutic outcomes.

VWF and ADAMTS13 levels may serve as prognostic and monitoring biomarkers in HCC across various therapeutic modalities. Surgical resection remains a primary curative approach, and these biomarkers could assist in predicting recurrence and vascular complications [73]. The outcomes of radiotherapy may be affected by VWF’s involvement in endothelial function and thrombosis [74]. Somatostatin analogs may influence angiogenesis through VWF modulation, presenting potential combination strategies [75]. Immunotherapy, particularly checkpoint inhibitors, may be informed by VWF and ADAMTS13 levels due to their association with immune cell dynamics [76]. Further research is warranted to incorporate these biomarkers into personalized treatment strategies.

This study may provide significant insights into the roles of *VWF* and *ADAMTS13* in HCC; however, there are several limitations of this study that warrant consideration. The findings were derived from a limited cohort with restricted geographic and demographic diversity, potentially compromising generalizability. The heavy reliance on public bioinformatics databases, which may introduce inherent biases, and the absence of comprehensive clinical variables, such as comorbidities and treatment histories, constrain the depth of the analysis. Experimental validation was not conducted and other regulatory mechanisms, including post-translational modifications, were not explored. Moreover, the study’s cross-sectional design and limited focus on tumor microenvironment complexity and functional miRNA/lncRNA interactions restrict its scope.To enhance the robustness and clinical relevance of the findings, future research should address these limitations through experimental studies, particularly those utilizing real-time polymer chain reaction (PCR) methodologies, as well as through the inclusion of diverse cohorts and longitudinal analyses.

## 5. Conclusions

The discrepant results obtained from the TCGA and GEO databases regarding VWF and ADAMTS13 highlight the imbalance between VWF and ADAMTS13 in hepatocellular carcinoma (HCC). Multiple factors may contribute to this imbalance, including alterations in methylation profiles tumor microenvironment and the effects of miRNAs and lncRNAs. Consequently, a thorough evaluation of these mechanisms is necessary. Although these findings are promising, they underscore the complexity of the interaction between VWF and ADAMTS13 in HCC progression. Further research should aim to validate these results in diverse and larger cohorts, as well as investigate the therapeutic potential of targeting these pathways. This study provides a foundation for the development of novel biomarkers and treatment strategies to advance the understanding of this challenging malignancy.

## Figures and Tables

**Table 1:** Combined score results of gene–gene interactions.

Gene-1	Gene-2	Protein Annotation	Combined Score
*VWF*	*ADAMTS13*	Von Willebrand antigen 2	0.997
*F8*	*ADAMTS13*	Factor VIIIa heavy chain	0.950
*GP1BA*	*ADAMTS13*	Platelet glycoprotein Ib alpha chain	0.931
*CFH*	*ADAMTS13*	Complement factor H	0.853
*CFHR1*	*ADAMTS13*	Complement factor H-related protein 1	0.833
*CFHR3*	*ADAMTS13*	Complement factor H-related protein 3	0.824
*HP*	*ADAMTS13*	Haptoglobin alpha chain	0.773
*DGKE*	*ADAMTS13*	Diacylglycerol kinase epsilon	0.767
*C3*	*ADAMTS13*	Complement C3c alpha’ chain fragment 1	0.735
*CFI*	*ADAMTS13*	Complement factor I heavy chain	0.721
*ITGA2B*	*VWF*	Integrin alpha-IIb light chain	0.999
*SELP*	*VWF*	Selectin P	0.999
*GP1BA*	*VWF*	Platelet glycoprotein Ib alpha chain	0.999
*F8*	*VWF*	Factor VIIIa heavy chain	0.999
*GP9*	*VWF*	Platelet glycoprotein IX	0.998
*FN1*	*VWF*	Fibronectin	0.998
*GP1BB*	*VWF*	Platelet glycoprotein Ib beta chain	0.998
*ITGB3*	*VWF*	Integrin beta-3; Integrin alpha-V/beta-3	0.998
*GP6*	*VWF*	Platelet glycoprotein VI	0.997
*ADAMTS13*	*VWF*	A disintegrin and metalloproteinase with thrombospondin motifs 13	0.997

**Table 2:** The miRNAs associated with *ADAMTS13* and *VWF* with a combination of TargetScanHuman8.0 and miRDB databases.

Predicted miRNAs for ADAMTS13	
miRDB databases TargetScanHuman8.0	hsa-miR-7850-5p, hsa-miR-4520-2-3p, hsa-miR-4516, hsa-miR-4434, hsa-miR-1263, hsa-miR-4462, hsa-miR-5703, hsa-miR-596, hsa-miR-3978, hsa-miR-6848-5p, hsa-miR-6846-5p, hsa-miR-3148
Predicted miRNAs for VWF	
miRDB databases TargetScanHuman8.0	hsa-miR-4296, hsa-miR-4322, hsa-miR-4265, hsa-miR-6759-5p, hsa-miR-6796-5p, hsa-miR-1972, hsa-miR-4437, hsa-miR-4468, hsa-miR-2278, hsa-miR-450b-5p, hsa-miR-2467-3p, hsa-miR-3190-5p

**Table 3:** *ADAMTS13* enrichment analysis result.

Term	Library	*p*-Value	q-Value	z-Score	Combined Score
thrombotic thrombocytopenic purpura	Jensen_DISEASES	0.00065	0.002275	19,987	146,700
Weill–Marchesani syndrome	Jensen_DISEASES	0.00065	0.002275	19,987	146,700
peptide catabolic process (GO:0043171)	GO_Biological_Process_2021	0.00125	0.00795	19,975	133,500
glycoprotein metabolic process (GO:0009100)	GO_Biological_Process_2021	0.00275	0.00795	19,945	117,600
organonitrogen compound catabolic process (GO:1901565)	GO_Biological_Process_2021	0.00315	0.00795	19,937	114,800
integrin-mediated signaling pathway (GO:0007229)	GO_Biological_Process_2021	0.00375	0.00795	19,925	111,300
peptide metabolic process (GO:0006518)	GO_Biological_Process_2021	0.00415	0.00795	19,917	109,200
thrombocytopenia	Jensen_DISEASES	0.00475	0.01108	19,905	106,500
protein maturation (GO:0051604)	GO_Biological_Process_2021	0.00485	0.00795	19,903	106,100
cell–matrix adhesion (GO:0007160)	GO_Biological_Process_2021	0.005	0.00795	19,900	105,400
protein processing (GO:0016485)	GO_Biological_Process_2021	0.0053	0.00795	19,894	104,200
diarrhea	Jensen_DISEASES	0.00755	0.01321	19,849	96,990
extracellular structure organization (GO:0043062)	GO_Biological_Process_2021	0.0108	0.01302	19,784	89,590
external encapsulating structure organization (GO:0045229)	GO_Biological_Process_2021	0.01085	0.01302	19,783	89,490
cerebrovascular disease	Jensen_DISEASES	0.01125	0.01575	19,775	88,740
proteolysis (GO:0006508)	GO_Biological_Process_2021	0.01435	0.015	19,713	83,660
RP11-395B7.4-ASO_G0227053_04-DEGs down	FANTOM6_lncRNA_KD_DEGs	0.01445	0.0539	19,711	83,520
hypertension	Jensen_DISEASES	0.01445	0.01686	19,711	83,520
extracellular matrix organization (GO:0030198)	GO_Biological_Process_2021	0.015	0.015	19,700	82,730
MEG3-ASO_G0214548_AD_09-DEGs Up	FANTOM6_lncRNA_KD_DEGs	0.0243	0.0539	19,514	72,540
CD27-AS1-ASO_G0215039_01-DEGs Down	FANTOM6_lncRNA_KD_DEGs	0.0246	0.0539	19,508	72,280
AC017048.3-ASO_G0163364_AD_01-DEGs Up	FANTOM6_lncRNA_KD_DEGs	0.02695	0.0539	19,461	70,330
CD71+ early erythroid	Human_Gene_Atlas	0.02765	0.0309	19,447	69,780
liver	Human_Gene_Atlas	0.0309	0.0309	19,382	67,390
ERVK3-1-ASO_G0142396_AD_07-DEGs Up	FANTOM6_lncRNA_KD_DEGs	0.0338	0.05408	19,324	65,460
RP11-473M20.14-ASO_G0263072_06-DEGs down	FANTOM6_lncRNA_KD_DEGs	0.0695	0.09267	18,610	49,620
RP11-458D21.1-ASO_G0233396_07-DEGs Up	FANTOM6_lncRNA_KD_DEGs	0.1262	0.1443	17,475	36,160
RAB30-AS1-ASO_G0246067_AD_06-DEGs down	FANTOM6_lncRNA_KD_DEGs	0.1552	0.1552	16,895	31,470
carcinoma	Jensen_DISEASES	0.5659	0.5659	8682	4943

**Table 4:** *VWF* enrichment analysis result.

Term	Library	*p*-Value	q-Value	z-Score	Combined Score
Factor XIII deficiency	Jensen_DISEASES	0.0003	0.002012	19,994	162,200
Hemostasis (GO:0007599)	GO_Biological_Process_2021	0.00035	0.00315	19,993	159,100
Congenital afibrinogenemia	Jensen_DISEASES	0.0004	0.002012	19,992	156,400
Factor VIII deficiency	Jensen_DISEASES	0.00045	0.002012	19,991	154,100
Factor XI deficiency	Jensen_DISEASES	0.00045	0.002012	19,991	154,100
Hemophilia B	Jensen_DISEASES	0.00055	0.002012	19,989	150,000
Thrombotic thrombocytopenic purpura	Jensen_DISEASES	0.00065	0.002012	19,987	146,700
Bernard–Soulier syndrome	Jensen_DISEASES	0.00065	0.002012	19,987	146,700
Intermittent claudication	Jensen_DISEASES	0.0007	0.002012	19,986	145,200
Purpura	Jensen_DISEASES	0.00085	0.00207	19,983	141,300
Von Willebrand’s disease	Jensen_DISEASES	0.0009	0.00207	19,982	140,100
Glanzmann’s thrombasthenia	Jensen_DISEASES	0.00105	0.002195	19,979	137,000
Diabetic retinopathy	Jensen_DISEASES	0.0019	0.003642	19,962	125,100
Vasculitis	Jensen_DISEASES	0.0029	0.005131	19,942	116,500
Complement and coagulation cascades	KEGG_2021_Human	0.00425	0.01547	19,915	108,800
ECM-receptor interaction	KEGG_2021_Human	0.0044	0.01547	19,912	108,000
Thrombocytopenia	Jensen_DISEASES	0.00475	0.007803	19,905	106,500
Platelet activation	KEGG_2021_Human	0.0062	0.01547	19,876	101,000
Platelet degranulation (GO:0002576)	GO_Biological_Process_2021	0.00625	0.0189	19,875	100,900
Pancreatic cancer	Jensen_DISEASES	0.00685	0.0105	19,863	98,990
Regulated exocytosis (GO:0045055)	GO_Biological_Process_2021	0.009	0.0189	19,820	93,360
Neutrophil extracellular trap formation	KEGG_2021_Human	0.00945	0.01547	19,811	92,350
Focal adhesion	KEGG_2021_Human	0.01005	0.01547	19,799	91,080
Coronary artery disease	Jensen_DISEASES	0.01025	0.01473	19,795	90,670
Extracellular structure organization (GO:0043062)	GO_Biological_Process_2021	0.0108	0.0189	19,784	89,590
External encapsulating structure organization (GO:0045229)	GO_Biological_Process_2021	0.01085	0.0189	19,783	89,490
Cerebrovascular disease	Jensen_DISEASES	0.01125	0.01522	19,775	88,740
Coronavirus disease	KEGG_2021_Human	0.0116	0.01547	19,768	88,100
Positive regulation of signal transduction (GO:0009967)	GO_Biological_Process_2021	0.0126	0.0189	19,748	86,380
Hypertension	Jensen_DISEASES	0.01445	0.01846	19,711	83,520
Lung	Human_Gene_Atlas	0.01495	0.01495	19,701	82,800
Extracellular matrix organization (GO:0030198)	GO_Biological_Process_2021	0.015	0.01929	19,700	82,730
Human papillomavirus infection	KEGG_2021_Human	0.01655	0.0177	19,669	80,670
PI3K-Akt signaling pathway	KEGG_2021_Human	0.0177	0.0177	19,646	79,260
Breast cancer	Jensen_DISEASES	0.0217	0.0261	19,566	74,950
Regulation of intracellular signal transduction (GO:1902531)	GO_Biological_Process_2021	0.02185	0.02458	19,563	74,800
Skin cancer	Jensen_DISEASES	0.0227	0.0261	19,546	73,990
Melanoma	Jensen_DISEASES	0.02525	0.02765	19,495	71,720
Positive regulation of intracellular signal transduction (GO:1902533)	GO_Biological_Process_2021	0.0273	0.0273	19,454	70,050
Kidney cancer	Jensen_DISEASES	0.1292	0.1351	17,416	35,640
Carcinoma	Jensen_DISEASES	0.5659	0.5659	8682	4943

## Data Availability

The data used in this study were obtained from the public database TCGA and others.

## Supplementary Materials

The following supporting information can be downloaded at: https://www.mdpi.com/article/10.3390/cimb47040270/s1.
